# Extracellular Vesicles of Adipose Multipotent Mesenchymal Stromal Cells Propagate Senescent Phenotype by Affecting PTEN Nuclear Import

**DOI:** 10.3390/ijms26157164

**Published:** 2025-07-24

**Authors:** Elizaveta Chechekhina, Semyon Kamenkov, Vadim Chechekhin, Anna Zinoveva, Elizaveta Bakhchinyan, Anastasia Efimenko, Natalia Kalinina, Vsevolod Tkachuk, Konstantin Kulebyakin, Pyotr Tyurin-Kuzmin

**Affiliations:** Medical Research and Educational Institute, Lomonosov Moscow State University, 119234 Moscow, Russiavadimchex97@gmail.com (V.C.); efimenkoan@gmail.com (A.E.);

**Keywords:** mesenchymal stem cells, cellular senescence, SASP, extracellular vesicles, PTEN nuclear import, insulin resistance, microRNAs

## Abstract

Replicative or stress-induced senescence disrupts the functioning of multipotent mesenchymal stromal cells (MSCs) required for tissue renewal and regeneration. Aged MSCs demonstrate reduced proliferation, impaired differentiation, and aberrant secretory activity, defined as “senescence-associated secretory phenotype” (SASP). SASP is characterized by elevated secretion of proinflammatory cytokines and specific extracellular vesicles (SASP-EVs), which affect the cellular microenvironment and promote tissue dysfunction. However, molecular mechanisms responsible for senescent phenotype propagation remain largely obscure. Earlier, we demonstrated suppression of adipogenic differentiation and insulin sensitivity of young MSCs by SASP-EVs. In this study, we elucidated potential mechanisms underlying SASP-EVs’ effects on MSCs. Bioinformatic analysis revealed that insulin signaling components are the most probable targets of SASP-EVs microRNA cargo. We demonstrated that SASP-EVs downregulated intracellular AGO1 levels, but surprisingly, PTEN levels were upregulated. Specifically, the increase in PTEN content was provided by its nuclear fraction. We have found that the intracellular PTEN distribution in young MSCs treated by SASP-EVs was similar to senescent MSCs. Furthermore, PTEN upregulation was accompanied by increased *PTENP1* expression—a molecular sponge for *PTEN*-targeting microRNAs. Our findings indicate that nuclear PTEN could be a hallmark of senescent MSCs, and SASP-EVs propagate the senescent phenotype in young MSCs by promoting PTEN nuclear localization.

## 1. Introduction

Aging-associated tissue dysfunction contradicts society’s cherished dream of longevity. In adipose tissue, aging is linked with impaired generation of new adipocytes and disturbed hormone-producing activity of mature adipocytes, which together play a central role in the pathogenesis of metabolic syndrome and associated disorders [1]. Adipocyte turnover relies on the activity of multipotent mesenchymal stromal cells (MSCs), a heterogeneous population, which harbors early adipocyte precursors as well as components of their microenvironment [2,3].

Age-related decline in the regenerative potential of MSCs has been reported in a number of studies [4]. Senescent MSCs demonstrate reduced proliferation and differentiation efficiency, indicating a gradual loss of their plasticity with aging, as well as altered ability to provide a microenvironment favorable for physiological tissue renewal [5]. For example, in aged adipose tissue, MSCs exhibit aberrant insulin signaling and signs of insulin resistance, which disrupts their ability for adipogenic differentiation [6].

Furthermore, MSCs isolated from adipose tissue of aged donors or undergoing senescence in vitro are characterized by profound changes in their secretome, demonstrating the so-called senescence-associated secretory phenotype (SASP). Such a phenotype is characterized by the shift towards the production of proinflammatory growth factors, secretion of a high quantity of extracellular vesicles [7,8], as well as a change in their microRNA content [9].

In our previous study [6], we demonstrated that SASP-associated EVs (SASP-EVs) impair the adipogenic differentiation of young MSCs. Therefore, SASP-EVs could play a central role in the development of age-related adipose tissue dysfunction and associated diseases.

This study was aimed at revealing a molecular mechanism underlying the effect of SASP-EVs on young MSCs, and the role of their microRNA in particular. We have demonstrated that SASP-EVs contain an elevated number of copies of microRNA, whose foreground predicted targets are insulin signaling pathway components. Surprisingly, these vesicles caused the elevation of PTEN content in young MSCs and induced its nuclear import. We have demonstrated that high PTEN expression and its nuclear localization attribute to aged MSCs. These data allowed us to suggest that SASP-EVs propagate the senescent phenotype within the MSC population by inducing PTEN nuclear import in young cells.

## 2. Results

### 2.1. SASP-EVs Carry microRNAs Regulating Insulin Signaling and Adipogenesis

**Figure 1:**
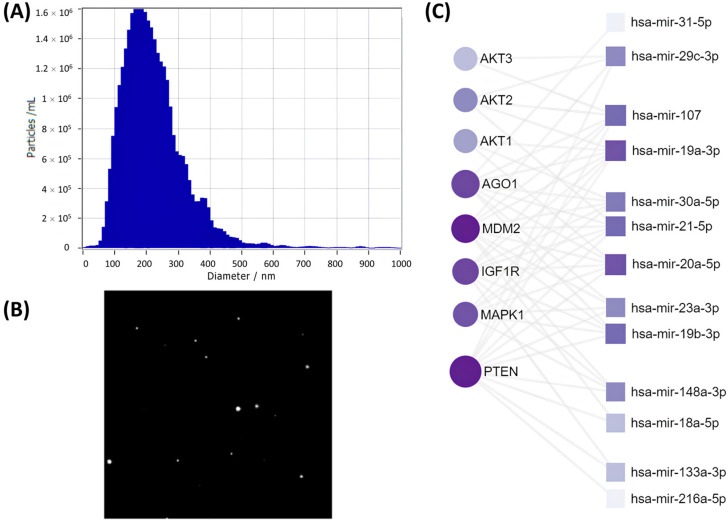
Characterization of SASP-EVs and their microRNA cargoes. (**A**) Quality control: size distribution of EVs determined by nanoparticle tracking analysis (NTA). (**B**) Representative screenshot from NTA video illustrating EVs’ movement. (**C**) Interaction map between microRNAs upregulated in the SASP and their target mRNAs. It displays connections between microRNAs uniquely increased in EVs from senescent MSCs and their corresponding targets. The size of the squares (microRNAs) and circles (their target mRNAs) indicates the number of interactions, while the color represents the strength of the interactions—darker colors correspond to stronger interactions.

To reveal a potential mechanism by which SASP-EVs impair the adipogenic differentiation capacity of young MSCs, we have characterized vesicle production by senescent cells as well as analyzed their microRNA cargoes. Nanoparticle tracking analysis (NTA) confirmed the isolation of EVs from the secretome of senescent MSCs with the expected size distribution (Figure 1A) and captured their Brownian motion dynamics (Figure 1B). Comparative microRNA profiling (Supplementary Table S1) revealed a distinct microRNA signature in SASP-EVs, characterized by 22 oligos significantly upregulated compared to EVs from young MSCs. Notably, bioinformatic analysis indicated that predicted mRNA targets of 13 of these microRNAs encode key components of the insulin signaling pathway, including Akt (a central kinase in insulin signaling), PTEN (a phosphatase that antagonizes PI3K/Akt activation), IGF1R (insulin-like growth factor 1 receptor), and MDM2 (a regulator of insulin receptor substrate stability). Notably, AGO1—a regulator of adipogenic differentiation and microRNA processing—was also identified as a key target. The interaction network (Figure 1C) highlights these interactions and their relative strengths. These findings directly link the microRNA cargoes of SASP-EVs to pathways critical for insulin sensitivity and adipogenesis.

### 2.2. SASP-EVs Alter the Expression of Insulin-Dependent Signaling Pathway Components

**Figure 2:**
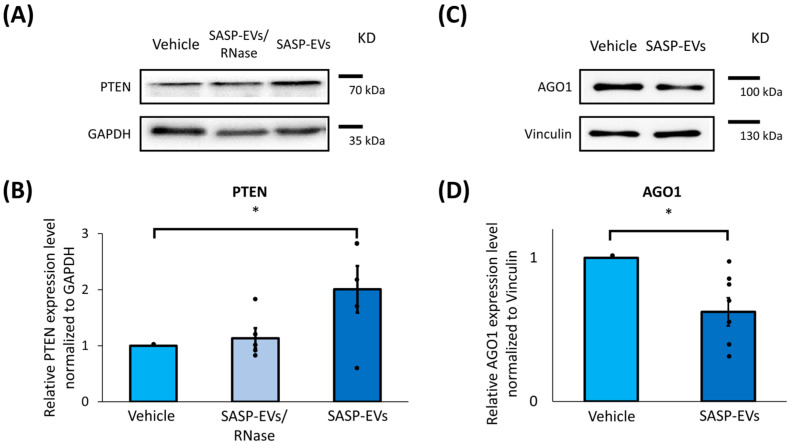
SASP-EVs affect the content of insulin signaling cascade components in young MSCs. (**A**,**B**) PTEN protein levels in young MSCs after EVs treatment, representative images (**A**), and quantification of band intensity (**B**). Data represent the mean ± SE, n = 5 (points), 5 donors, * *p* < 0.05. (**C**,**D**) AGO1 protein levels in young MSCs after EVs treatment, representative images (**C**), and quantification of band intensity (**D**). Vehicle—culture medium without EVs; SASP-EVs—vesicles isolated from the secretome of senescent MSCs; SASP-EVs/RNase—vesicles isolated from the secretome of senescent MSCs and pre-treated by RNase before application. Data represent the mean ± SE, n = 7 (points), 5 donors, * *p* < 0.05.

To analyze the effect of SASP-EVs on predicted components of the insulin-dependent signaling pathway, we examined protein levels in young MSCs and confirmed that all identified targets—Akt, PTEN, AGO1, MDM2, and IGF1R—were expressed in untreated cells (Figure 2, Supplementary Figures S1–S3). Notably, treatment with SASP-EVs led to an unexpected twofold increase in PTEN protein content (Figure 2A,B), a result that stands in contrast to the anticipated microRNA-mediated suppression and highlights a non-canonical regulatory effect. Our bioinformatic search has revealed over 10 miRNAs targeting PTEN (Figure 1). To perform a robust assessment of the role of RNA transfer in PTEN translocation, we have treated isolated EVs by RNase before the application to cells. We have demonstrated that the effect of EVs on PTEN was abolished by RNase treatment (Figure 2A,B). In line with expectations, AGO1 levels decreased significantly following SASP-EV exposure (Figure 2C,D). Meanwhile, the protein levels of Akt, MDM2, and IGF1R remained largely unchanged (Supplementary Figure S1). However, SASP-EVs caused upregulation of Akt phosphorylation. These findings indicate a complex modulatory effect of SASP-EVs on insulin signaling components in young MSCs.

### 2.3. SASP-EVs Promote PTEN Nuclear Import in Young MSCs, Recapitulating the Senescence-Associated PTEN Localization

**Figure 3:**
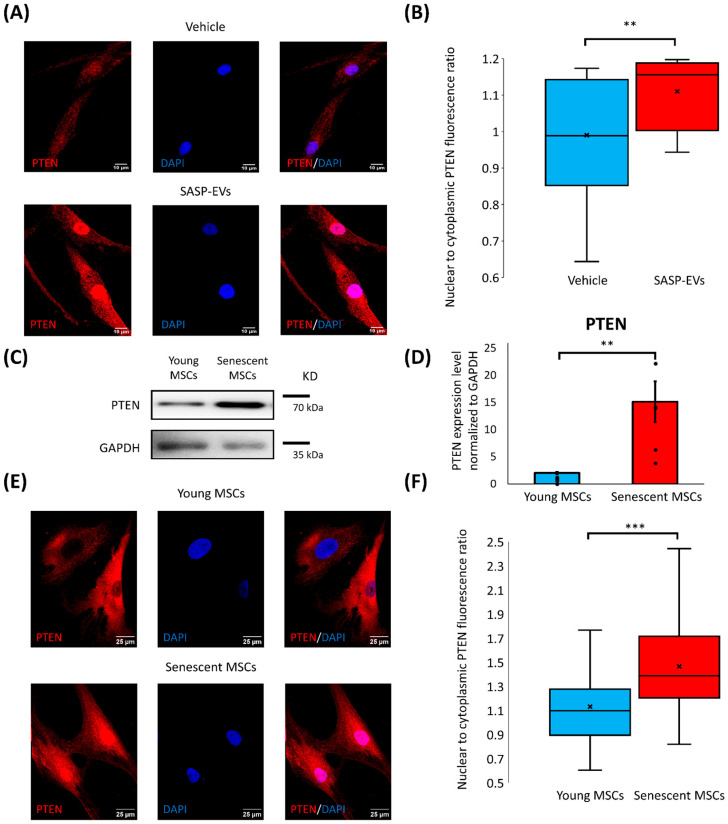
SASP-EVs induce PTEN nuclear import in young MSCs. (**A**) Immunofluorescent images of PTEN localization in young MSCs without and treated with senescent EVs. (**B**) Quantification of fluorescence intensity of immunofluorescent images. The distribution of data with median (line), mean (cross), and interquartile range, n = 19–20 individual cells, 2 donors, ** *p* < 0.01. (**C**,**D**) PTEN protein levels in young and senescent MSCs, representative images (**C**), and quantification of band intensity (**D**). Data represent the mean ± SE, n = 5–6 (points), 11 donors, ** *p* < 0.01. (**E**) Immunofluorescent images of PTEN localization in young and senescent MSCs. (**F**) Quantification of fluorescence intensity of immunofluorescent images. The distribution of data with median (line), mean (cross), and interquartile range, n = 124–348 individual cells, 7 donors, *** *p* < 0.001. (**A**,**E**) In the immunofluorescent images, PTEN appears in red, while nuclei are stained with DAPI (blue). For the control staining with non-specific IgG, please refer to Supplementary Figure S6.

To further investigate the mechanisms responsible for the regulation of PTEN, we considered the fact that the functions of this protein depend on its subcellular localization. Therefore, we assessed PTEN distribution between the nucleus and cytoplasm in young MSCs before and after treatment by SASP-EVs. We have found that SASP-EVs caused a significant increase in nuclear PTEN in recipient young MSCs (Figure 3A,B), indicating that the elevation of PTEN is largely attributable to its nuclear accumulation.

To better understand whether the PTEN nuclear import caused by SASP-EVs is related to the senescent phenotype of vesicle-producing MSCs, we next examined the expression and subcellular distribution of PTEN in senescent MSCs themselves.

Indeed, we have demonstrated markedly higher total PTEN protein levels in MSCs from aged patients compared to young cells (Figure 3C,D). This upregulation was accompanied by the increased level of nuclear PTEN (Supplementary Figure S4). Consistent with this, immunofluorescent staining revealed a pronounced nuclear localization of PTEN in senescent MSCs (Figure 3E,F). Furthermore, we have analyzed whether replicative senescence is also accompanied by nuclear PTEN upregulation. Indeed, in long-term cultures (11–12 passages), MSCs contain an elevated amount of PTEN in the nucleus (Supplementary Figure S5).

Together, these findings suggest that nuclear accumulation of PTEN is a hallmark of senescent MSCs’ phenotype, and SASP-EVs induce a phenotypic shift in young MSCs, making them recapitulate a feature observed in senescent cells.

### 2.4. PTEN Nuclear Localization in Senescent MSCs Is Associated with Elevated PTENP1 Levels

Since we observed the unexpected overexpression of PTEN in response to SASP-EVs, we hypothesized that compensatory mechanisms protect *PTEN* mRNA from the repression caused by vesicle-derived microRNAs (Figure 1C). Thus, non-coding RNAs, including pseudogenes, can act as molecular decoys that sequester microRNAs, thereby preventing microRNA-mediated repression of target mRNAs.

**Figure 4:**
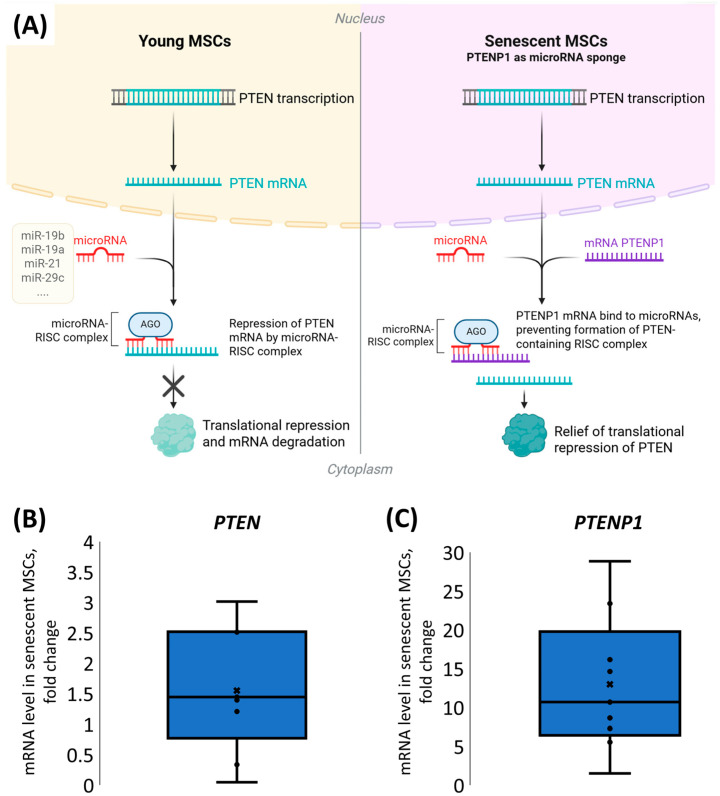
Analysis of *PTEN* and *PTENP1* mRNA levels in young and senescent MSCs. (**A**) Mechanism of *PTENP1* sequestration of microRNAs targeting *PTEN*. (**B**) Change in *PTEN* mRNA level in senescent MSCs. (**C**) Change in *PTENP1* mRNA expression level in senescent MSCs. (**B**,C) mRNA expression was evaluated using the ΔΔCt method. The distribution of data is shown with individual data points, median (line), mean (cross), and interquartile range, n = 6–8, 8 donors.

For the *PTEN* gene, a unique pseudogene, *PTENP1*, exists, which is known to sequester *PTEN* mRNAs targeting microRNAs (Figure 4A). To elucidate whether *PTENP1* contributes to the sustained high levels of PTEN protein observed in senescent MSCs, we assessed its expression during cellular senescence. We have demonstrated that *PTENP1* mRNA expression was dramatically elevated, on average by 15-fold (Figure 4B,C), whereas *PTEN* mRNA levels showed only a modest increase in senescent MSCs compared to young cells.

Taken together, these data indicate that elevated expression of the *PTENP1* pseudogene in senescent MSCs may act as a molecular “sponge” for anti-*PTEN* microRNAs and therefore is responsible for the elevated PTEN protein level and its nuclear import in senescent MSCs (Figure 4A).

## 3. Discussion

The secretome of MSCs from various tissue sources is considered an attractive cell-free therapeutic approach suggested for the treatment of severe disorders, including autoimmune conditions, fibrosis, and chronic inflammation [10,11,12]. This study has revealed a molecular mechanism responsible for the potential detrimental effect of MSCs’ secretome components, which can sufficiently compromise their therapeutic applications.

Our study demonstrates that SASP-EVs alter the expression of insulin signaling pathway components, which correlates well with changes in their microRNA content. Specifically, SASP-EVs are enriched in microRNAs targeting components of the insulin signaling pathway, which is consistent with our previous report [6]. Both replicative and stress-induced MSC senescence were associated with the specific changes in vesicle cargoes. These SASP-EVs could be involved in the paracrine-dependent induction of insulin resistance. It is important to note that the large-scale manufacturing of MSC-derived EVs requires a sufficient amplification of the producing cells. This can ultimately lead to the accumulation of senescent cells, especially in primary (not immortalized) cultures, and therefore to the increase in the SASP-EVs ratio in the preparations of vesicles designated to be used as therapeutics.

Among predicted SASP-EVs microRNAs targets, *PTEN* was the most prominently affected. We found a remarkable upregulation of total PTEN level in young MSCs upon treatment with SASP-EVs, which was accompanied by its nuclear import. Cytoplasmic PTEN was previously reported to be involved in the regulation of stem cells’ quiescence, differentiation, and senescence by interfering with Notch signaling, the PTEN/PI3K/Akt pathway, and other intracellular cascades [13]. Nuclear PTEN is studied in detail in cancer cells, where it contributes to genomic stability maintenance and cell cycle arrest [14], but this is the first report, to our knowledge, that nuclear PTEN is suggested to be involved in stem cell senescence. SASP-EVs suppress adipogenic differentiation of young MSCs [6] and activate PTEN nuclear import at the same time. This correlation of impaired adipogenic capacity of MSCs [6] with PTEN nuclear translocation allows us to speculate that nuclear PTEN can interfere with MSC plasticity. Nuclear PTEN functioning—protective or detrimental—as well as molecular mechanisms controlling its translocation in MSCs, clearly requires further elucidation.

This study highlights the nuclear accumulation of PTEN as an early hallmark of senescent cells, which can be used to evaluate the number of MSCs developing the senescent phenotype. Our data indicate that SASP-EVs propagate the senescent phenotype, at least in part, by influencing PTEN content and nuclear import in young MSCs. In tissues, this mechanism could lead to the accumulation of senescent cells and exacerbate tissue dysfunction during aging, further compromising its regenerative potential. Senescent MSC accumulation in adipose tissue could compromise adipocyte turnover and lead to the development of metabolic disorders.

While increased microRNA levels typically lead to downregulation of their target proteins [15,16,17], PTEN demonstrated a marked upregulation in response to SASP-EVs. This paradoxical increase strongly suggests the involvement of other regulatory interactions. We demonstrated that senescent cells demonstrate remarkably elevated expression of *PTENP1*, a pseudogene that acts as a molecular sponge for microRNAs repressing *PTEN* mRNA [18,19]. This high *PTENP1* expression could be responsible for sustained high PTEN protein levels in senescent MSCs despite the elevated expression of its target microRNAs.

In conclusion, PTEN nuclear import is associated with the senescent phenotype of MSCs, and SASP-EVs readily propagate this phenotype. We suggest that our data provide a basis for the development of quality control tests for EV-based therapeutics. On the other hand, further elucidation of nuclear PTEN functioning in MSCs will clarify the regulatory networks governing PTEN localization and expression in aging MSCs, which is essential for the development of efficient strategies to combat age-related decline in tissue function.

### Study Limitations

The major limitation of this study is a lack of functional validation of nuclear PTEN’s role in senescence. Also, the search for specific miRNAs, which mediate PTEN nuclear translocation, will further clarify suggested mechanisms. Finally, the hypothesis that *PTENP1* sponging protects *PTEN* mRNA from SASP-EV miRNAs needs to be pursued further.

## 4. Materials and Methods

### 4.1. Cell Culture

Primary MSC cultures were obtained from abdominal adipose tissue samples of healthy young donors (n = 10, median age 36 years) and aged donors (n = 8, median age 67.5 years, age > 65). These cell lines were sourced from the biobank at the Institute for Regenerative Medicine, Medical Research and Education Center, Lomonosov Moscow State University (collection ID: MSU_MSC_AD). All experiments followed the Declaration of Helsinki guidelines and were approved by the Ethics Committee of Lomonosov Moscow State University (IRB00010587, protocol #4, 4 June 2018, and protocol #9, 29 October 2018). Donors provided informed consent prior to sample collection. MSCs were cultured in a Mesenchymal Stem Cell Basal Medium enriched with 10% Mesenchymal Stem Cell Growth Supplement, 1% Penicillin/Streptomycin, and 1% L-glutamine. Cells were maintained at 37 °C in a 5% CO_2_ atmosphere and were passaged when they reached 70–80% confluence, using Versen and HyQTase solutions, with a subculturing ratio of 1:3. For experiments, MSCs from young and aged donors were used at passages 4–5. Also, senescent MSCs were characterized as described in our previous paper [6].

### 4.2. Extracellular Vesicle Isolation

EVs were isolated from MSCs obtained from aged donors (age-related senescent MSCs at passages 2–5) and young donors (MSCs at passages 2–5) and handled in compliance with the current MISEV 2023 guidelines [20]. Cells were cultured until they reached 90–100% confluence, then washed three times with Hank’s buffer and incubated in low-glucose DMEM without serum and phenol red for 48 h. The conditioned medium was collected, centrifuged at 300× *g* for 10 min to remove debris, and EVs were isolated by ultrafiltration using Sartorius filters (1000 kDa), as previously published [6]. EV samples were stored at −80 °C. Their size and concentration were measured via nanoparticle tracking analysis (NTA), their morphology was visualized using transmission electron microscopy (TEM), and exosomal markers were evaluated by immunoblotting.

### 4.3. MicroRNA Analysis from Extracellular Vesicles and Bioinformatics

To investigate microRNAs secreted by young and senescent MSCs, we isolated these molecules using the miRNeasy Mini Kit (Qiagen, Hilden, Germany) according to the manufacturer’s guidelines. RNA quality and quantity were assessed using a Nanodrop spectrophotometer (Thermo Scientific, Waltham, MA, USA) by evaluating the 260/230 nm ratio. Reverse transcription was conducted with the miScript II RT Kit (Qiagen), following the recommended protocol. Real-time PCR was performed using specific miScript miRNA PCR Arrays (Qiagen) and the miScript SYBR Green PCR Kit (Qiagen), which included 2x QuantiTect SYBR Green PCR Master Mix and a universal miRNA primer on the QuantStudio 5 Real-Time PCR System (Thermo Fisher Scientific). The expression levels of miRNAs were calculated relative to those of housekeeping miRNAs (SNORD61 variants, SNORD95, SNORD96a, and RNU6) using the comparative ΔCT method.

We analyzed the microRNA array data using the sRNAtoolbox web-server (version 0.0.4) with parameters set to miRBase v22 for microRNAs, Ensembl release 91 for non-coding RNAs, a seed length of 20, two allowed mismatches, and a phred score of 20. To identify microRNAs associated with senescence, we utilized databases such as TargetScan7.2, HMDD, miR2Disease, and miRwayDB. Functional predictions of microRNAs were made using the Gene Ontology (GO) and Kyoto Encyclopedia of Genes and Genomes (KEGG) databases. GO and KEGG gene clustering was performed with the David 6.8 database. Quality control, mapping, and normalization of the microRNA array data were conducted using the BrowserGenome 1.0 web-based platform (hg38 GENCODE 22 genome). Keyword analysis was performed using custom bash scripts involving commands like while read line, grep, awk, sed, and sort (uniq). Common predicted microRNA targets were analyzed using miRNet, miRBase, and miRDB databases.

After compiling lists of targets for identified microRNAs, we used the miRNet platform to create interaction maps. In these maps, microRNAs were represented as nodes, and their targets as edges. Also, in the diagram, the size of a node reflects the number of interactions with the target, whereas the intensity of shading indicates the strength of the interaction, with darker colors corresponding to more frequent interactions.

### 4.4. Nuclear and Cytoplasmic Protein Separation

To isolate nuclear and cytoplasmic protein fractions, cells were cultured until 70–80% confluency in 100 mm dishes. After washing twice with cold PBS, cells were detached using 0.25% trypsin-EDTA (Gibco, Waltham, MA, USA) and centrifuged at 300× *g* for 5 min. The resulting pellet was resuspended in 500 µL of hypotonic buffer (10 mM HEPES, pH 7.9, 10 mM KCl, 1.5 mM MgCl_2_, 0.5 mM DTT) supplemented with protease and phosphatase inhibitors (Sigma-Aldrich, St. Louis, MO, USA). Cells were incubated on ice for 10 min, followed by the addition of 0.3% Triton X-100 to permeabilize the plasma membrane. The cell suspension was vortexed for 10 s and centrifuged at 800× *g* for 5 min at 4 °C. The supernatant containing the cytoplasmic fraction was carefully collected and stored at −80 °C.

The nuclear pellet was resuspended in 300 µL of nuclear lysis buffer (20 mM HEPES, pH 7.9, 420 mM NaCl, 1.5 mM MgCl_2_, 0.2 mM EDTA, 25% glycerol, 0.5 mM DTT) with protease and phosphatase inhibitors. The suspension was homogenized and incubated on ice for 30 min, with intermittent vortexing. After incubation, the sample was centrifuged at 16,000× *g* for 15 min at 4 °C. The supernatant containing the nuclear protein fraction was collected and stored at −80 °C.

Protein concentrations in the cytoplasmic and nuclear fractions were measured using a Bradford assay (Bio-Rad, Hercules, CA, USA), and the fractions were subjected to downstream analyses such as Western blotting. Fraction integrity was confirmed by detecting β-actin (cytoplasmic marker) and histone H3 (nuclear marker) in respective samples.

### 4.5. Western Blotting

For Western blotting, proteins were extracted from MSC lysates by cell lysis in sample buffer. Protein separation was performed using SDS-PAGE, followed by transfer to a PVDF membrane. Non-specific binding was blocked with a solution of TBS containing 0.1% Tween 20 and 5% BSA. Membranes were incubated overnight with primary antibodies targeting PTEN (Abcam, Cambridge, UK, ab267787), IGF1R (Cell Signalling, Danvers, MA, USA, D23H3), MDM2 (Abcam, ab16895), AGO1 (Abcam, ab300152), Akt (Cell Signaling, 9272S), Vinculin (Sigma, St. Louis, MO, USA, V4139), GAPDH (Cell Signalling, D16H11), Histone H3 (Cell Signaling, D1H2), and beta-actin (Cell Signaling, 4970S) as a loading control. Following washes, membranes were incubated with secondary HRP-conjugated antibodies (Goat Anti-Mouse IgG Antibody, Sigma-Aldrich; Goat Anti-Rabbit IgG Antibody, Sigma-Aldrich). Chemiluminescence detection was conducted using a Clarity ECL detection kit (Bio-Rad, Hercules, CA, USA), and images were captured using the Clinx gel documentation system. Quantitative analysis was performed using Image Lab version 6.0.1.

### 4.6. Immunofluorescence Staining of Cell Cultures

Immunofluorescence staining was performed as follows: cells were fixed in 10% buffered formalin for 10 min at room temperature. Permeabilization was carried out using a 0.2% Triton X-100 solution for 10 min, followed by two washes with phosphate-buffered saline (PBS). To minimize nonspecific binding, cells were incubated for 30 min in a blocking solution containing 1% bovine serum albumin (BSA) and 10% goat serum (donor of secondary antibodies). After removing excess blocking solution, cells were incubated for 1 h with primary antibodies against PTEN (Rabbit Recombinant Monoclonal PTEN antibody, Abcam, ab267787) diluted 1:100. Cells were washed three times with PBS and then incubated for 1 h with secondary antibodies (Goat anti-rabbit Alexa Fluor 594, Invitrogen, Waltham, MA, USA, 1:500) and DAPI (Sigma, 1:10,000). Following three washes with PBS, cells were mounted in an aqueous medium (Aqua-Polymount, Polysciences, Warrington, PA, USA). As a negative control, rabbit non-immune IgG was used in place of primary antibodies.

This protocol efficiently stains PTEN while minimizing background fluorescence, allowing for clear nuclear and cytoplasmic imaging.

### 4.7. Reverse-Transcription Quantitative PCR (RT-qPCR)

RNA was extracted from MSCs using the RNeasy Mini Kit (Qiagen, Hilden, Germany), and cDNA was synthesized from 500 ng of RNA using the MMLV Reverse Transcription Kit (Eurogene, Moscow, Russia). Quantitative real-time PCR was performed to analyze the expression of PTEN and PTENP1, using SYBR Green-based detection. PCR amplification was carried out on a CFX96 Touch Real-Time PCR System (Bio-Rad, Hercules, CA, USA), with 60S Ribosomal protein P0 (*RPLP0*) used as a reference gene. Gene expression was quantified using the ΔΔCT method, and primer sequences were selected using the NCBI Primer Designing Tool (version 2.5.0).

We used 60S Ribosomal protein P0 (*RPLP0*), For: GCTGCTGCCCGTGCTGGTG, Rev: TGGTGCCCCTGGAGATTTTAGTGG, 130 bp. *PTEN*, For: TTTGAAGACCATAACCCACCAC, Rev: ATTACACCAGTTCGTCCCTTTC, 134 bp. *PTENP1*, For: TCAGAACATGGCATACACCAA, Rev: TGATGACGTCCGATTTTTCA, 153 bp.

### 4.8. Statistical Analysis

Data were analyzed using SigmaPlot 12.5 software. The normality of distribution was assessed using the Shapiro–Wilk test. Results are presented as mean ± SEM or medians with interquartile ranges. For comparisons between two independent groups, Student’s *t*-test was used for normally distributed data, while the Mann–Whitney U test was applied for non-normally distributed data. For multiple group comparisons, the Kruskal–Wallis test (one-way ANOVA on ranks) followed by Dunn’s post hoc test was performed. Statistical significance was set at *p* < 0.05.

## Data Availability

Data are contained within the article and Supplementary Materials. The data presented in this study are available on request from the corresponding author.

## Supplementary Materials

The following supporting information can be downloaded at: https://www.mdpi.com/article/10.3390/ijms26157164/s1.

## Abbreviations

The following abbreviations are used in this manuscript:

MSCs	Mesenchymal stem cells
SASP	Senescence-associated secretory phenotype
EVs	Extracellular vesicles
SASP-EVs	SASP-associated extracellular vesicles
