# Supplementary material for: Extracellular Vesicles of Adipose Multipotent Mesenchymal Stromal Cells Propagate Senescent Phenotype by Affecting PTEN Nuclear Import
Source: Int J Mol Sci. 2025 Jul 24;26(15):7164. doi: 10.3390/ijms26157164 (PMC12345871; doi:10.3390/ijms26157164)
Supplement: Supplementary file 1 [file ijms-26-07164-s001.zip › ijms-3746449-supplementary.pdf]

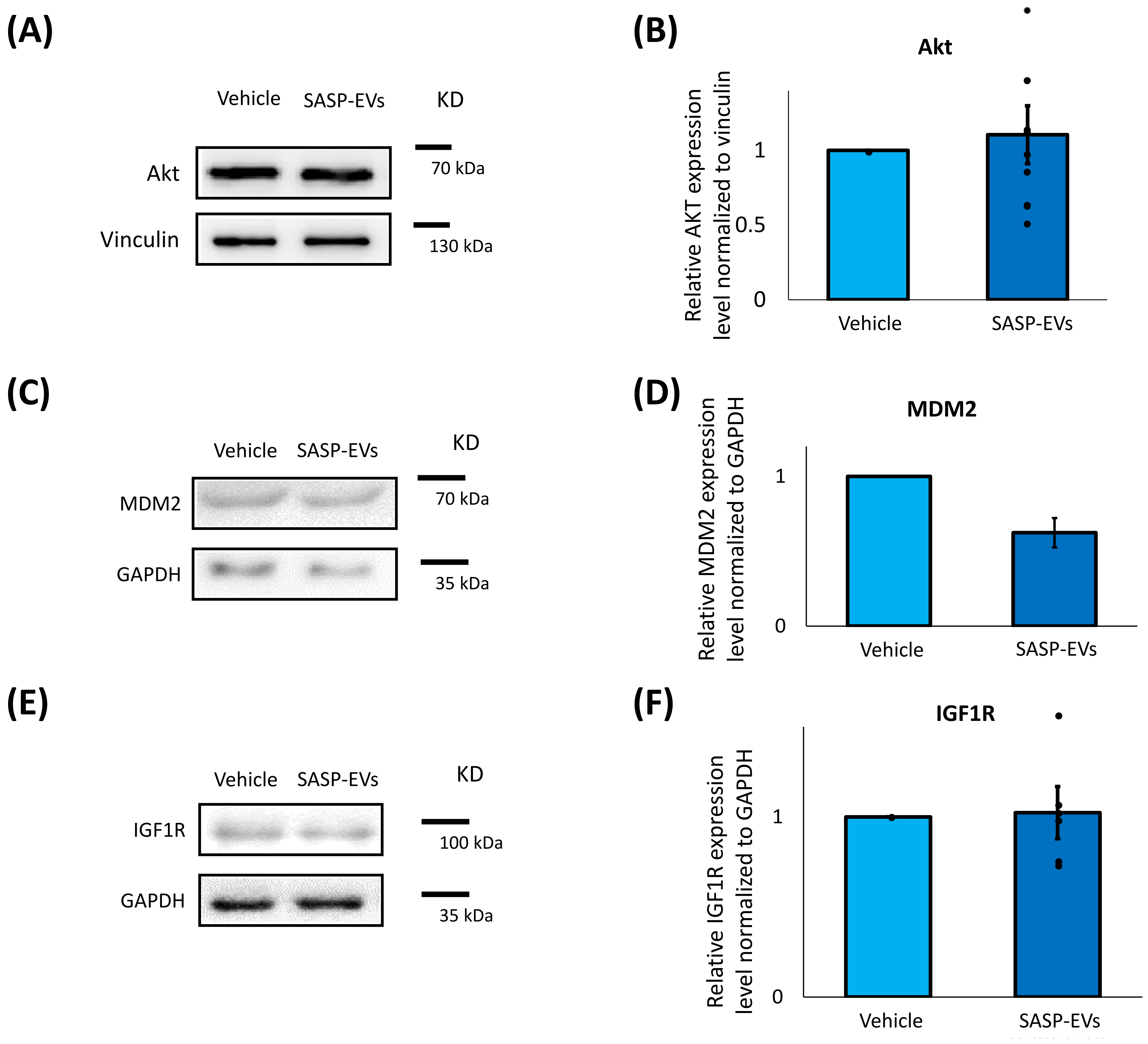

Figure S1. SASP-EVs affect the content of insulin signaling cascade components in young MSCs. (A-B) Akt protein levels in young MSCs after EVs treatment, representative images (A), and quantification of band intensity (B). Data represent the mean ± SE, n = 10 (points), 5 donors. (C-D) MDM2 protein levels in young MSCs after EVs treatment, representative images (C), and quantification of band intensity (D). Data represent the mean ± SE, n = 3 (points), 3 donors. (E-F) IGF1R protein levels in young MSCs after EVs treatment, representative images (E), and quantification of band intensity (F). Data represent the mean ± SE, n = 6 (points), 6 donors.

(A)

Western blot analysis of PTEN expression

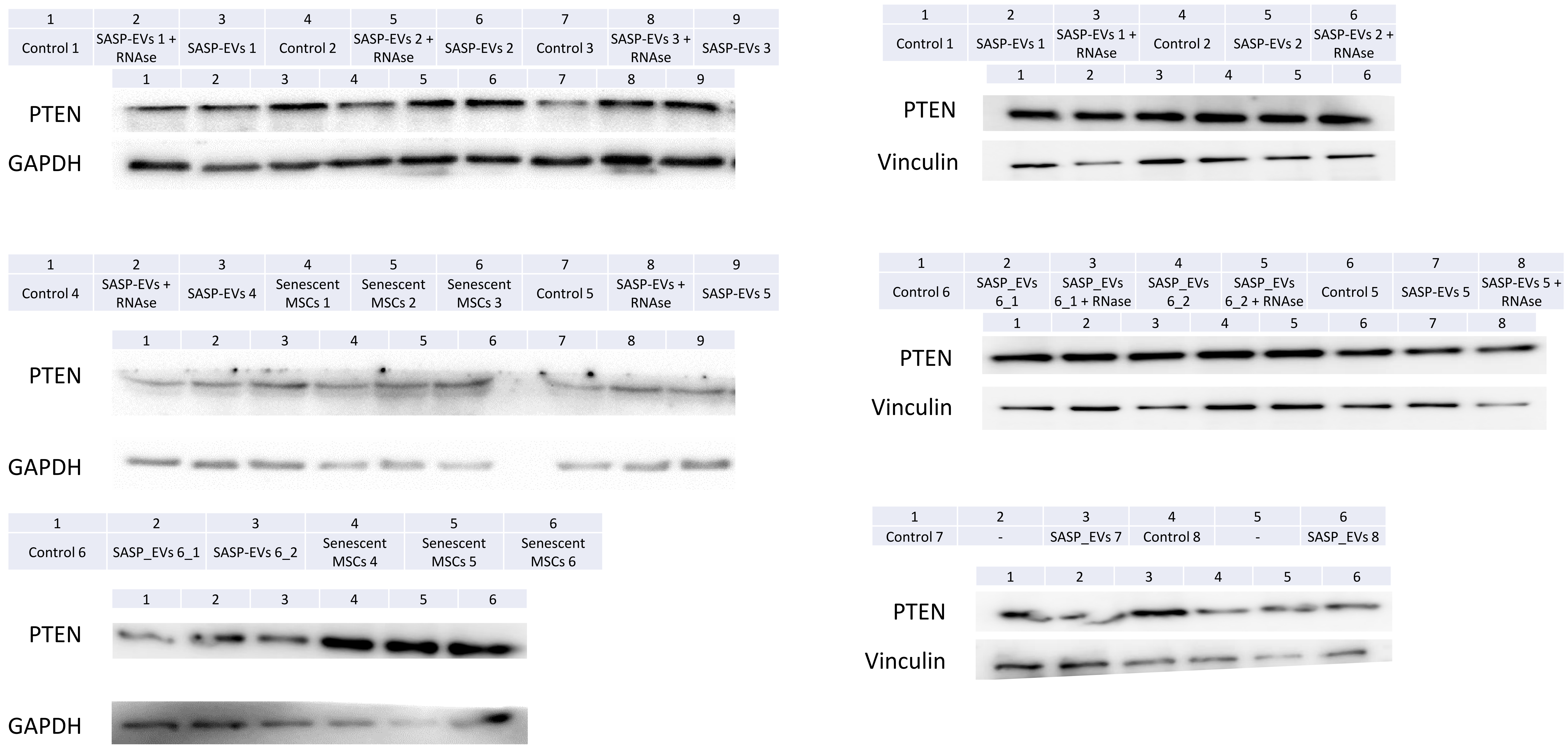

(B)

Western blot analysis of Ago1 expression

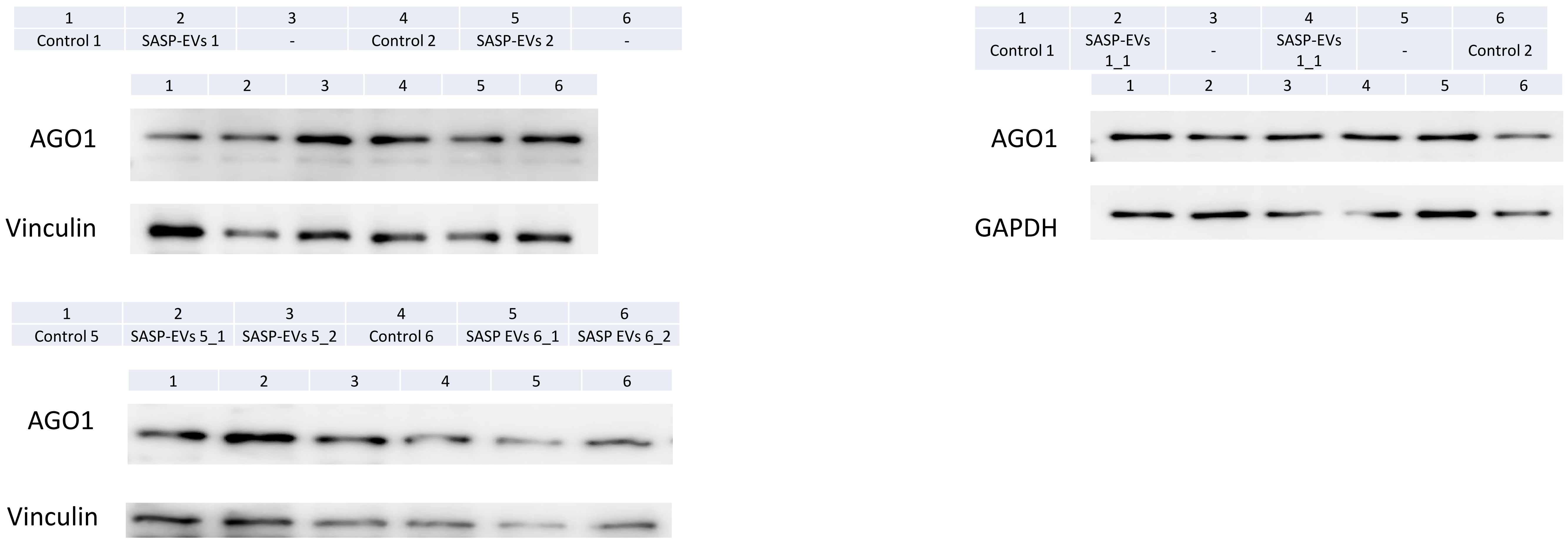

Figure S2. Results of Western-blot analysis of PTEN (A) and Ago1 (B) in young MSCs, young MSCs after SASP-EVs treatment and senescent MSCs (for PTEN only).

(A)

Western blot analysis of Akt expression

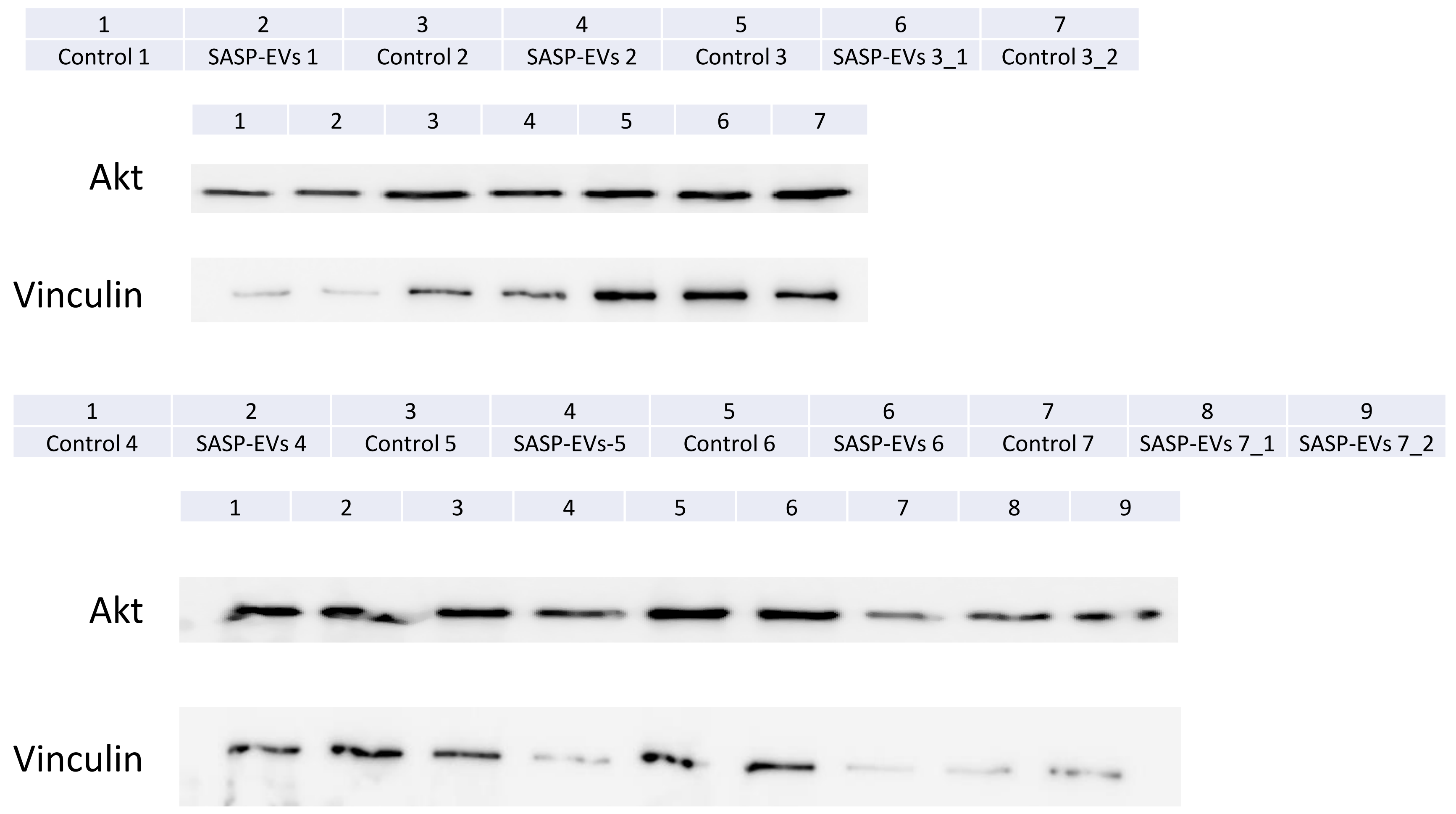

(B)

Western blot analysis of IGF1R expression

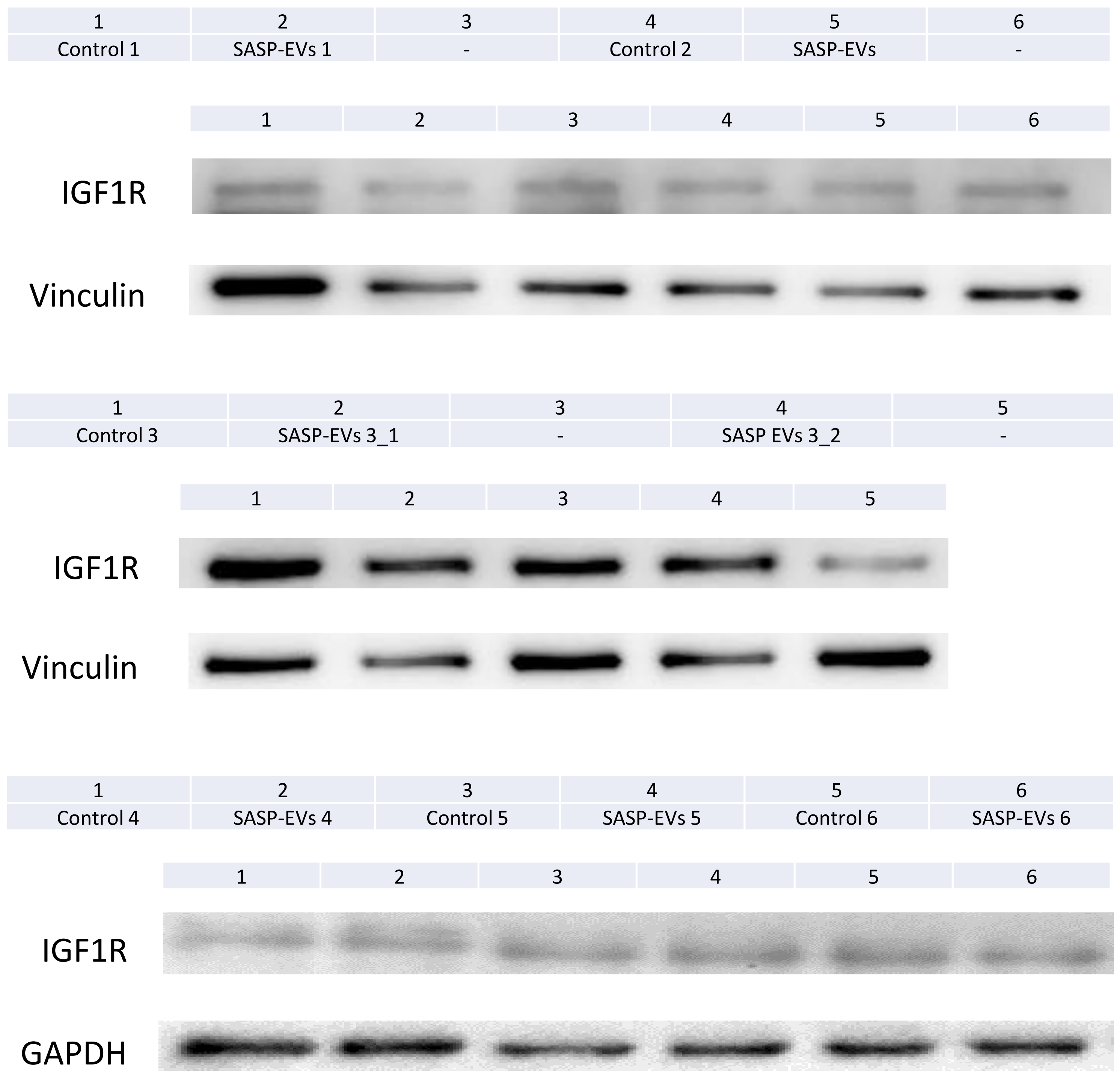

(C)

Western blot analysis of MDM2 expression

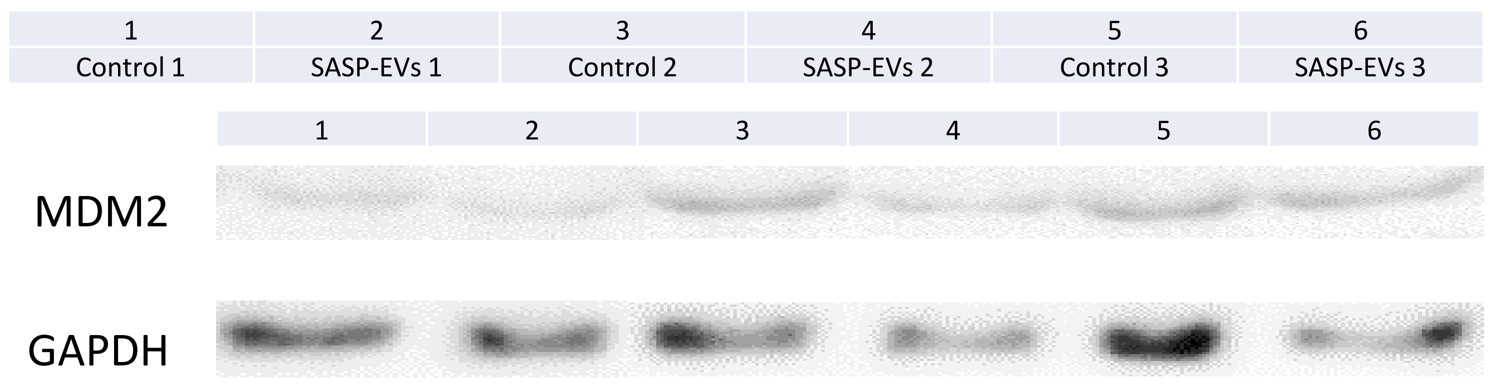

Figure S3. Results of Western-blot analysis of Akt (A), IGF1R (B) and MDM2 (C) in young MSCs and young MSCs after SASP-EVs treatment.

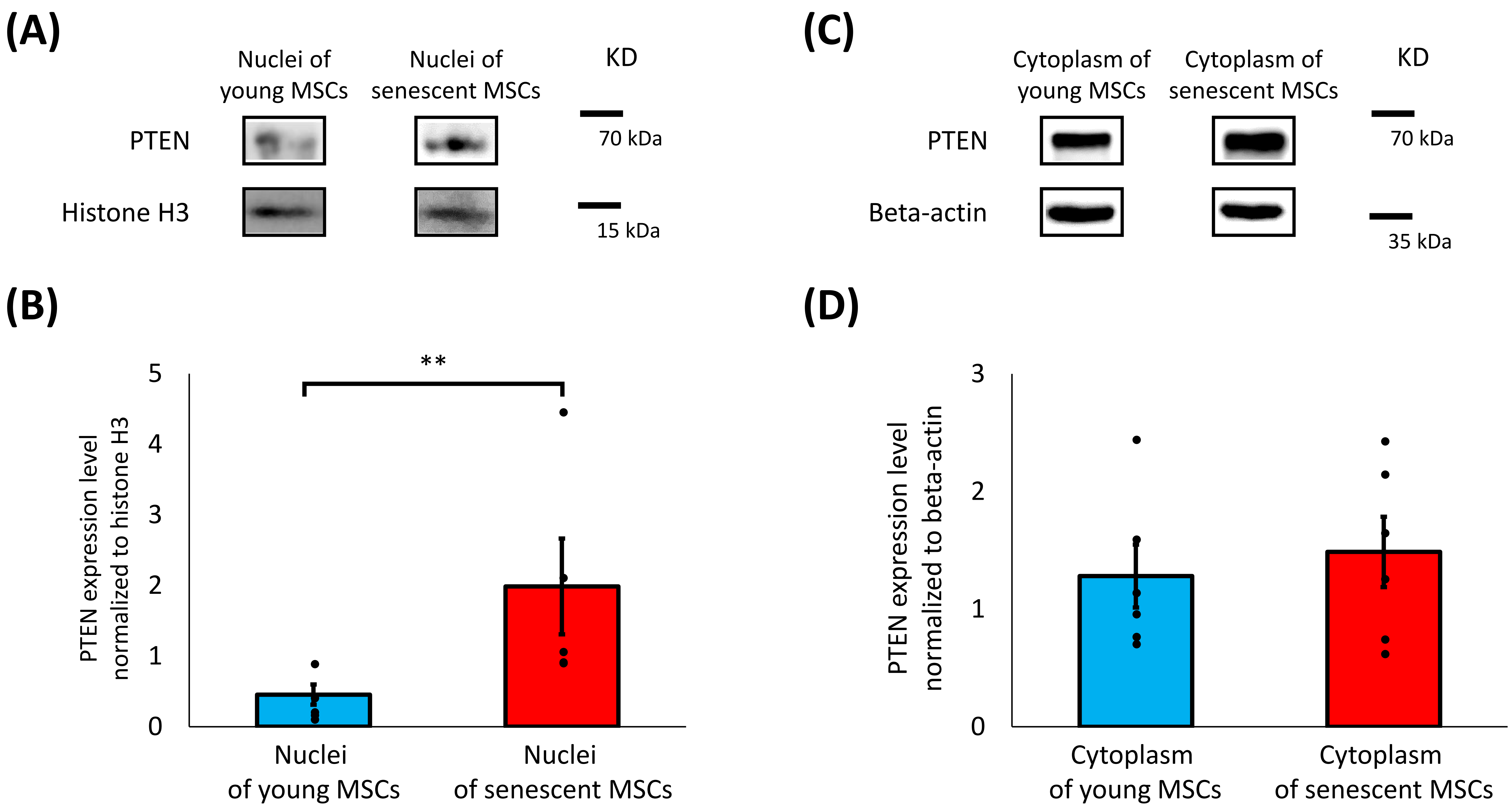

Figure S4. Nuclear accumulation of PTEN in senescent MSCs. (A-B) Western blot analysis of nuclear fractions of PTEN in young and senescent MSCs, representative images (A), and quantification of bands intensity (B). (C-D) Western blot analysis of cytoplasmic fractions of PTEN in young and senescent MSCs, representative images (C), and quantification of bands intensity (D). (B-D). Data represent the mean ± SE, n = 4-6 (points), 9 donors, **$p < 0.01$.

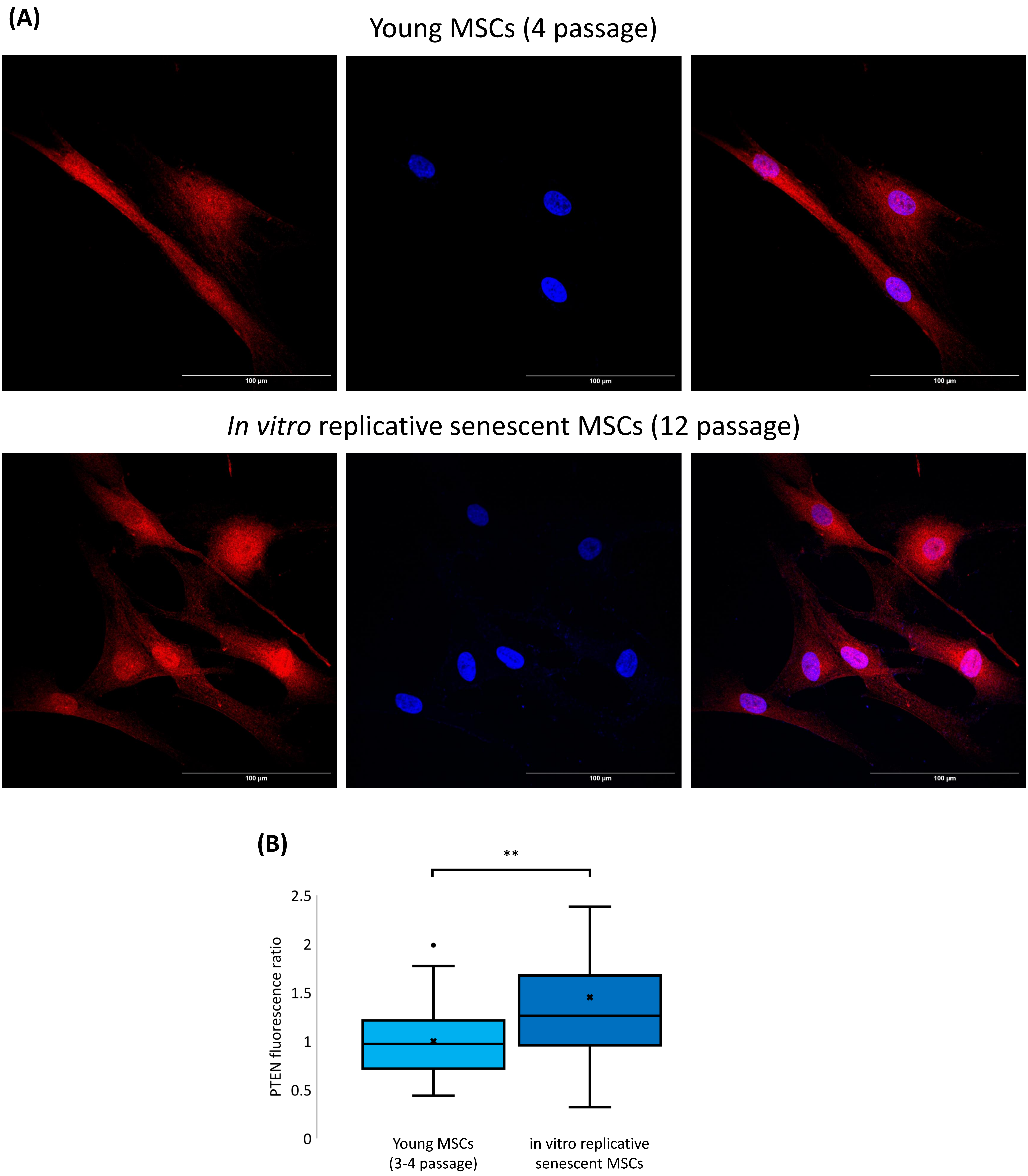

Figure S5. Replicative senescence leads to PTEN nuclear import in MSCs. (A) Immunofluorescent images of PTEN localization in 4 passage (young) and 12 passage (replicative senescent) MSCs from the same donor. (B) Quantification of fluorescence intensity of immunofluorescent images. The distribution of data with median (line), mean (cross), and interquartile range, n = 36 individual cells, 3 donors, **$p < 0.01$.

Immunocytochemistry

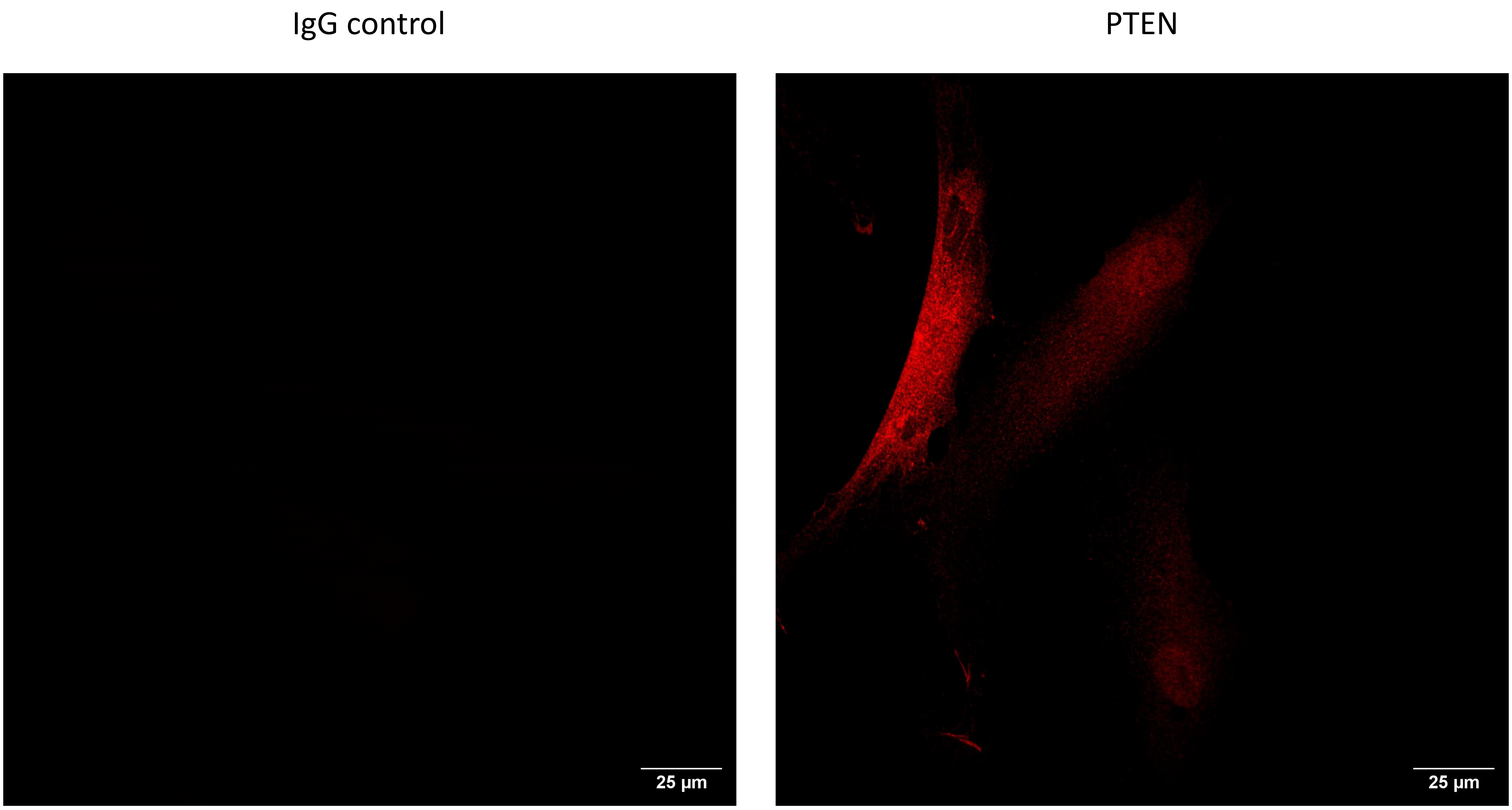

Figure S6. Immunofluorescent images of IgG control and PTEN staining.

Table S1. Analysis of microRNA sequencing in EVs.

Target	Fold-change EVs old vs. young	Target	Fold-change EVs old vs. young		
miR-200b-3p	0.000517469	miR-29c-3p	1.501049567		**Downregulated**
miR-451a	0.001800619	miR-30a-5p	1.54064051		**Upregulated**
miR-203a-3p	0.015900824	miR-21-5p	1.694376232		
miR-491-5p	0.019919199	miR-195-5p	1.806387117		
miR-126-3p	0.169705479	miR-23a-3p	1.890276724		
let-7d-5p	0.238665835	miR-20a-5p	2.026320142		
miR-215-5p	0.241471025	miR-196-5p	2.070626826		
miR-101-3p	0.255352251	miR-5011-5p	2.108184826		
miR-34a-5p	0.398297689	miR-216a-5p	2.269245013		
miR-155-5p	0.44133977	miR-143-3p	2.430231073		
miR-7-5p	0.44705297	miR-19b-3p	2.467972949		
miR-16-5p	0.452970674	miR-328-3p	2.790248424		
miR-129-5p	0.569678778	miR-199b-5p	2.853584981		
miR-146a-5p	0.580116498	miR-31-5p	2.87865517		
		miR-199a-5p	3.054907056		
**Only in SASP-EVs**		miR-146b-5p	3.401072608		
MiR-107		miR-194-5p	4.488065484		
miR-141-3p		miR-449a	4.754327017		
miR-148a-3p		miR-5692a	12.0612147		
miR-211-5p		miR-375	13.24782693		
miR-302b-3p		miR-449b-5p	13.61909238		
miR-377-3p		miR-18a-5p	34.99343231		
		miR-590-5p	35.89545238		
**Only in young**		miR-19a-3p	305.8166492		
miR-26a-5p		miR-133a-3p	645.328471		
